# Flow of Formulation Granules through a Conical Hopper

**DOI:** 10.4103/0250-474X.49133

**Published:** 2008

**Authors:** B. Mazumder, R. Rajan, Jasmina Khanam, A. Nanda

**Affiliations:** Department of Pharmaceutical Technology, Jadavpur University, Kolkata-700 032, India

**Keywords:** Conical-hopper, dimensional analysis, flowability, granules

## Abstract

Gravity flow characteristics of various pharmaceutical granules through static conical hoppers of different cone angles were studied. Mass flow rate depends on properties of granules and cone angles when environmental conditions such as temperature and relative humidity are kept within a fixed range. The granules were made with active pharmaceutical ingredients as per Indian pharmacopoeia with other additives like binders and diluents. Lubricants were added with the granules to observe their effects on mass flow rate. Magnesium stearate and colloidal silicon dioxide of different proportions were used as lubricants after granulation. A new dimensionally analyzed equation was developed to predict flow rate of the granules. The developed equation agreed well with the experimental data with a percentage deviation of ±10%.

The parameters, which control powder flowability through cylindrical and conical hoppers, are numerous. In an earlier paper^1^, it was shown that the discharge rate of granules through a cylindrical hopper depends on average granule diameter, apparent density of the granules, orifice diameter, orifice diameter correction factor, coefficient of friction (equivalent to granule-granule shear stress), acceleration due to gravity and density as well as viscosity of air at the operating temperature. Using dimensional analysis method, a correlation was established as Q.(μ')^−0.6^/(d_p_.μ.ρ_p_/ρ)=0.0004.{(d_p_)^3^.(ρ_p_)^2^.g/μ^2^}^0.45^.{d_p_/(d_o_-kd_p_)}^−2.5^, where Q is theoretical flow rate of granules, μ´ is coefficient of friction, d_p_ is particle diameter, μ is viscosity of air, ρ_p_ is apparent density of granules, ρ density of air, g is acceleration due to gravity, d_o_ is hopper orifice diameter, kd_p_ is orifice diameter correlation factor.

Other parameters like drag on granules per unit area (R_T_), granule-wall shear stress (R_w_) and void fraction (ɛ) were not taken into consideration during dimensional analysis because R_T_ depends on d_p_, ρ_p_, g, ρ and μ; R_w_ was negligible as the cylindrical hoppers were made of acrylic and ɛ depends on kd_p_. Bulk density (ρ_b_) was not taken into consideration because ρ_b_ varies with respect to both time and position within the hopper. Flow of granules through conical hopper had been investigated by several workers. Deming and Mehring^2^ proposed a discharge rate equation relating d_o_, d_p_, ρ_p,_. μ´ and θ with Q. Peter York^3^ studied the effect of glidents on flowablity of cohesive pharmaceutical powders. Gold *et al*^4^ studied the effect of several glidants on mass discharge rate and angle of repose (α). No correlation was proposed by them, but optimum amounts of glidants to be added to increase the free flowing nature of the granules through conical hopper were predicted. Flow of pharmaceutically important granules at lower pressure (10 psi) in a stainless steel conical hopper having interchangeable orifices was also studied by Gold *et al*^5^. Easy flow of poorly flowing granules was reported but they did not present any mathematical equation for calculating the discharge rate. Gravity flow of granular materials from conical bunker at positive pressure of air was studied by Holland *et al*^6^. They incorporated the term ‘d_p_/d_r_’ and ɛ in the final correlation for determining granule velocity at the orifice. The pressure gradient acts in the radial direction and combines with the gravitational force to enhance the discharge rate.

A new flow model to predict gravity flow of soya bean meal of different sizes through conical hoppers of different angles of 40°, 50° and 60° and with different orifice sizes of 3.175 cm to 8.89 cm was developed by Wang *et al*^7^. The variables opted for dimensional analysis were diameter of the hopper (D), orifice diameter (d_o_), acceleration due to gravity (g), apparent density of granules (ρ_p_), bulk density of the granules (ρ_b_), hopper angle (2θ), angle of repose (α) and Q. They predicted a generalized correlation which is Q=C. ρ_b._ d_0_^2.5^.√g.(d_o_/D)^m^. (α/θ)^n^, where C, m and n are constants. They presented three sets of values for C, m and n on different moisture contents of the feed and concluded that the correlation is useful in predicting the discharge rate at the smallest meal sizes but not suitable for higher meal sizes.

Earlier investigators on conical hopper flow did not consider the influence of the properties of air, like ρ and μ on the discharge rates of the pharmaceutical granules. Some of them considered the influence of air pressure on the discharge rate during the flow of granules at higher-pressure condition but others neglected the influence of atmospheric air on the flow rate when the flow of granules was carried out through conical hopper at normal pressure. The orifice diameter correction factor ‘kd_p_’ is not considered during the present investigation because there is no existence of stagnation zones of granules at the bottom and around the orifice of a conical hopper made of glass (negligible R_w_), which however may exist during the flow of powder through a cylindrical hopper of any make. The objectives of the present investigation are to (i) study the flow properties of formulation granules in conical hoppers (ii) develop equation correlating the flow variables (iii) compare theoretical mass flow rate with actual values.

Indian Pharmacopoeia grade chemicals/drugs like calcium carbonate, famotidine, dicalcium phosphate, acetyl salicylic acid, metronidazole, ofloxacin, norfloxacin, paracetamol, sulphamethoxazole, trimethoprim, diltiazem hydrochloride, amoxicillin, diclofenac sodium, nimesulide, ranitidine, doxycycline, mebendazole, starch, polyvinyl pyrrolidone, talc, magnesium stearate and colloidal silicon dioxide (aerosil) were obtained as gift samples from reputed pharmaceutical concerns in Kolkata, India.

Fifteen sets of dried formulation granules were prepared. To prepare each set of granules, drugs and chemicals were wet granulated using necessary amount of starch or polyvinyl pyrrolidone paste as binder in a lab model planetary mixer and with standard ten-mesh sieve. Finally the wet granules thus prepared were dried in a tray drier to moisture content below 5% w/w. Talc (2% w/w) as glidant was mixed with dried granules in another planetary mixer. Each set of dried granules was then divided into 5 equal parts. One part was kept as normal granules (Composition of fifteen sets of normal granules is given in Table 1) and other four parts were lubricated separately as normal granules with 0.5% w/w magnesium stearate (A), normal granules with 0.5% w/w magnesium stearate and 0.5% w/w aerosil (B), normal granules with 1% w/w magnesium stearate and 0.5% w/w aerosil (C), normal granules with 1% w/w magnesium stearate and 1% w/w aerosil (D).

**TABLE 1:** COMPOSITION OF 15 SETS OF NORMAL GRANULES

Active Ingredient(s)	Inactive Ingredients
Calcium carbonate 95% w/w	starch
Famotidine 25% w/w, Dicalcium phosphate 65% w/w	starch
Acetyl salicylic acid 90% w/w	polyvinyl pyrrolidone
Metronidazole 87.5% w/w	starch
Ofloxacin 90% w/w	starch
Norfloxicin 95% w/w	polyvinyl pyrrolidone
Paracetamol 95% w/w	starch
Sulphamethoxazole 80% w/w, Trimethoprim 16% w/w	starch
Diltiazem hydrochloride 90% w/w	starch
Diclofenac sodium 62.5% w/w	starch
Nimesulide 90% w/w	starch
Amoxicillin 80% w/w	polyvinyl pyrrolidone
Ranitidine 90% w/w	polyvinyl pyrrolidone
Doxycycline 62.5% w/w	starch
Mebendazole 62.5% w/w	starch

Mean surface diameter (d_s_) of the dried granules in a part of a set was determined by sieve analysis method^8^. Apparent density of the granules was determined by specific gravity method. Angle of repose was measured by standard funnel heap method. Coefficient of friction (μ') = tanα = height of heap/radius of circle, where α is the angle of repose. Hopper cone angle (2θ) and orifice diameter (d_o_) were determined by standard physical methods.

Three conical hoppers made of borosilicate glass are of 25°, 32° and 38° included angles but of same d_o_ of 1.15 cm. Each hopper has two portions, a cylindrical portion (D=4.56 cm) at the top and the conical portion with the orifice at the bottom. To determine the flow rate of a particular part of granules of a set through a particular hopper the hopper was completely filled with the granules keeping the orifice closed by sliding disc of 0.5 cm thick and made of S.S. The method of filling granules into the hopper was of natural filling. The ratio of d_o_ and the highest d_s_ of the granules was above 5:1 (d_o_:d_s_>5:1); while the ratio of D and the highest d_s_ was more than 20:1 (D:d_s_>20:1). The ratio of D and d_o_ was nearly 4:1. The influence of head on flow rate is negligible when the material head height exceeds 2.5 D^1^,^7^. Therefore the head height was always maintained above 12 cm throughout the experiment. Flow rate was calculated by weighing the granules flowing through the orifice within a defined time interval. The flow rate was determined in triplicate and average value (Q') was tabulated.

After careful examining of the data and results of earlier investigations as well as considering the assumption and predictions taken in our earlier paper^1^, it is proposed that Q=f(d_s_, d_o_, ρ_p_, μ', gcosθ, ρ, μ) where d_s_ is mean surface diameter of granules. The variables are analyzed by dimensional analysis method using standard dimensions. The correlation obtained is, Q.(μ')^a^/(d_s_.μ)= A.{d_s_)^3^.(ρ_p_)^2^.gcosθ/μ^2^}^b^.(ρ_p_/ρ)^c^.(d_0_/d_s_)^d^…1. The constants A, a, b, c and d are determined by putting experimental values from Table 1 and experimental flow rates of granules from Table 2 in Eqn. 1. The final equation is: Q.(μ)^−0.6^/(d_s_.μ.ρ_p_/ρ)= 0.15.{d_s_)^3^.(ρ_p_)^2^.gcosθ/μ^2^}^0.45^. (d_o_/d_s_)^1.1^…2.

**TABLE 2:** PHYSICAL CHARACTERISTICS OF GRANULES WITH OPERATING CONDITIONS

Index	d_s_	ρ_p_	μ'	ρ* 10^3^	μ* 10^4^	t (°)	Index	d_s_	ρ_p_	μ'	ρ* 10^3^	μ* 10^4^	t (°)
1	0.198	1.772	0.606	1.184	1.78	25.0	9	0.082	1.581	0.622	1.186	1.77	24.5
1A	0.197	1.779	0.606	1.184	1.78	25.0	9A	0.082	1.587	0.566	1.186	1.77	24.5
1B	0.197	1.779	0.598	1.184	1.78	25.0	9B	0.082	1.594	0.560	1.186	1.77	24.5
1C	0.198	1.78	0.591	1.184	1.78	25.0	9C	0.081	1.599	0.551	1.186	1.77	24.5
1D	0.197	1.78	0.586	1.184	1.78	25.0	9D	0.081	1.619	0.546	1.186	1.77	24.5
2	0.098	1.725	0.571	1.186	1.77	24.5	10	0.058	1.624	0.531	1.180	1.78	26.0
2A	0.098	1.755	0.566	1.186	1.77	24.5	10A	0.057	1.643	0.517	1.180	1.78	26.0
2B	0.098	1.765	0.558	1.186	1.77	24.5	10B	0.058	1.695	0.512	1.180	1.78	26.0
2C	0.098	1.785	0.551	1.186	1.77	24.5	10C	0.056	1.725	0.491	1.180	1.78	26.0
2D	0.098	1.797	0.544	1.186	1.77	24.5	10D	0.057	1.753	0.493	1.180	1.78	26.0
3	0.064	1.802	0.521	1.153	1.83	33.0	11	0.200	1.480	0.606	1.184	1.78	25.0
3A	0.063	1.823	0.517	1.153	1.83	33.0	11A	0.199	1.488	0.604	1.184	1.78	25.0
3B	0.064	1.857	0.512	1.153	1.83	33.0	11B	0.201	1.494	0.600	1.184	1.78	25.0
3C	0.062	1.894	0.508	1.153	1.83	33.0	11C	0.200	1.450	0.596	1.184	1.78	25.0
3D	0.063	1.925	0.503	1.153	1.83	33.0	11D	0.200	1.513	0.590	1.184	1.78	25.0
4	0.188	1.702	0.598	1.186	1.77	24.5	12	0.214	1.685	0.756	1.190	1.77	23.5
4A	0.188	1.712	0.595	1.186	1.77	24.5	12A	0.213	1.705	0.749	1.190	1.77	23.5
4B	0.188	1.712	0.593	1.186	1.77	24.5	12B	0.214	1.715	0.744	1.190	1.77	23.5
4C	0.187	1.716	0.587	1.186	1.77	24.5	12C	0.215	1.752	0.736	1.190	1.77	23.5
4D	0.187	1.729	0.583	1.186	1.77	24.5	12D	0.212	1.798	0.731	1.190	1.77	23.5
5	0.145	1.382	0.545	1.184	1.78	25.0	13	0.143	1.514	0.735	1.184	1.78	25.0
5A	0.143	1.390	0.541	1.184	1.78	25.0	13A	0.139	1.529	0.734	1.184	1.78	25.0
5B	0.142	1.396	0.539	1.184	1.78	25.0	13B	0.136	1.531	0.733	1.184	1.78	25.0
5C	0.139	1.427	0.538	1.184	1.78	25.0	13C	0.144	1.536	0.732	1.184	1.78	25.0
5D	0.140	1.441	0.534	1.184	1.78	25.0	13D	0.132	1.543	0.728	1.184	1.78	25.0
6	0.166	1.446	0.583	1.190	1.77	23.5	14	0.108	1.384	0.711	1.153	1.83	33.0
6A	0.163	1.453	0.576	1.190	1.77	23.5	14A	0.109	1.406	0.705	1.153	1.83	33.0
6B	0.163	1.459	0.572	1.190	1.77	23.5	14B	0.107	1.426	0.696	1.153	1.83	33.0
6C	0.164	1.459	0.558	1.190	1.77	23.5	14C	0.110	1.467	0.692	1.153	1.83	33.0
6D	0.164	1.462	0.543	1.190	1.77	23.5	14D	0.107	1.498	0.682	1.153	1.83	33.0
7	0.210	1.48	0.642	1.190	1.77	23.5	15	0.104	1.188	0.648	1.153	1.83	33.0
7A	0.212	1.488	0.639	1.190	1.77	23.5	15A	0.101	1.196	0.643	1.153	1.83	33.0
7B	0.207	1.489	0.637	1.190	1.77	23.5	15B	0.096	1.238	0.636	1.153	1.83	33.0
7C	0.207	1.499	0.635	1.190	1.77	23.5	15C	0.102	1.265	0.632	1.153	1.83	33.0
7D	0.214	1.516	0.632	1.190	1.77	23.5	15D	0.101	1.276	0.625	1.153	1.83	33.0
8	0.166	1.501	0.631	1.190	1.77	23.5							
8A	0.165	1.509	0.630	1.190	1.77	23.5							
8B	0.165	1.517	0.627	1.190	1.77	23.5							
8C	0.168	1.525	0.621	1.190	1.77	23.5							
8D	0.166	1.533	0.605	1.190	1.77	23.5							

Physical characteristics of particles, as mentioned in method, have been tabulated in Table 2. Density of air (ρ) at an operating temperature (t) has been calculated on the assumption that air is an ideal gas and its viscosity μ at the operating temperatures are taken from standard literature. Operational temperature varies from 23.5° to 33°. Properties of air ρ and m were considered in framing Eqn. 2 since flow rate of granules through conical hopper depends on these factors even at the normal atmospheric conditions. Measurements of all the physical factors of each category of granules have been carried out in triplicate and average values were presented in Table 2. Bulk density (ρ_b_) is considered since the particle density (ρ_p_) within a hopper varies with respect to both time and position and flowing density (ρ_pf_, ratio of the mass flow rate to the volumetric flow rate) was found to be substantially independent of the initial voidage or the mass flow rate^6^.

Average experimental flow rate (Q') of each category of granules, calculated theoretical flow rate of the same category (Q) from the equation (2) and the percentage deviation [{Q'-Q)/Q'}×100] are tabulated in Table 3 (hopper angle 25°, 32° and 38°). From the data, it is clear that Q' increases with the degree of lubrication in each and every group of granules. It is also observed that Q' decreases with the increase of hopper angle in each and every category of granules. From Table 3, it is observed that the percentage deviation is within ±10%.

**TABLE 3:** ACTUAL DISCHARGE RATES (Q'), THEORETICAL DISCHARGE RATES (Q) WITH PERCENT DEVIATIONS

Index	25°			32°			38°		
			
	Q'	Q	%	Q'	Q	%	Q'	Q	%
1	21.25	22.28	−4.84	21.08	22.13	−4.94	21.05	21.96	−4.35
1A	21.54	22.37	−3.84	21.17	22.21	−4.93	21.55	22.05	−2.31
1B	21.81	22.20	−1.80	21.79	22.05	−1.17	21.72	21.88	−0.72
1C	22.67	22.09	2.57	22.58	21.94	2.85	22.18	21.94	1.83
1D	22.82	21.93	3.90	22.35	21.78	2.58	22.37	21.62	3.39
2	9.28	8.46	8.83	9.14	8.40	8.10	8.95	8.34	6.87
2A	9.48	8.67	8.54	9.48	8.61	9.11	9.28	8.55	7.85
2B	9.62	8.70	9.54	9.54	8.64	9.44	9.47	8.58	9.42
2C	9.76	8.83	9.54	9.59	8.77	8.53	9.50	8.70	8.35
2D	9.82	8.87	9.62	9.63	8.81	8.46	9.58	8.75	8.72
3	6.89	6.60	4.23	6.35	6.55	−3.15	5.98	6.50	−8.64
3A	6.87	6.65	3.29	6.78	6.60	2.70	6.13	6.55	−6.80
3B	7.18	6.96	3.08	7.04	6.91	1.86	6.24	6.86	−9.91
3C	7.48	6.94	7.19	7.27	6.90	5.14	6.32	6.85	−8.35
3D	7.89	7.37	6.59	7.80	7.32	6.15	6.68	7.26	−8.68
4	20.06	19.11	4.71	17.71	18.98	7.18	17.72	18.84	−6.29
4A	20.68	19.27	6.81	18.06	19.13	−5.96	18.00	18.99	−5.52
4B	20.85	19.26	7.65	18.47	19.12	−3.55	18.27	18.98	−3.93
4C	21.18	19.20	9.35	18.91	19.06	−0.83	18.49	18.92	−2.33
4D	21.38	19.38	9.36	19.18	19.24	−0.31	18.65	19.10	−2.39
5	8.77	8.39	4.34	8.66	8.33	3.83	7.56	8.27	−9.32
5A	8.95	8.46	5.47	8.72	8.40	3.70	7.92	8.34	−5.18
5B	9.12	8.42	7.65	8.97	8.36	6.82	8.00	8.30	−3.69
5C	9.35	8.49	9.20	9.18	8.43	8.15	8.12	8.37	−3.07
5D	9.40	8.71	7.36	9.33	8.64	7.36	8.15	8.58	−5.22
6	12.26	11.60	5.39	11.90	11.52	3.23	10.66	11.43	−7.18
6A	12.60	11.59	8.01	12.51	11.51	7.94	10.75	11.43	−6.31
6B	12.77	11.64	8.88	12.61	11.56	8.39	10.89	11.47	−5.36
6C	12.72	11.53	9.41	12.67	11.45	9.65	11.15	11.36	−1.91
6D	12.55	11.38	9.35	12.52	11.30	9.75	11.85	11.21	5.38
7	14.76	15.70	−6.36	14.71	15.59	−6.02	14.15	15.48	−9.35
7A	15.84	15.97	−0.82	15.07	15.86	−5.26	14.40	15.74	−9.31
7B	16.24	15.49	4.62	15.82	15.38	2.77	14.55	15.26	−4.90
7C	16.48	15.58	5.47	16.22	15.47	4.61	14.81	15.36	−3.69
7D	16.99	16.43	3.27	16.77	16.32	2.72	15.11	16.20	−7.19
8	13.68	13.32	2.60	12.22	13.23	−8.24	11.92	13.13	−10.15
8A	13.85	13.33	3.72	12.35	13.24	−7.23	11.99	13.14	−9.56
8B	13.99	13.39	4.27	12.39	13.29	−7.33	12.29	13.20	−7.35
8C	14.06	13.79	1.41	12.43	13.69	−10.12	12.36	13.59	−9.90
8D	14.12	13.52	4.22	12.89	13.43	−4.18	12.48	13.33	−6.76
9	5.65	6.04	−6.84	5.46	6.00	−9.96	5.42	5.96	−9.94
9A	5.89	5.79	1.84	5.54	5.75	−3.65	5.45	5.70	−4.70
9B	6.14	5.75	6.38	5.93	5.71	3.66	5.83	5.67	2.83
9C	6.21	5.70	8.28	6.15	5.66	8.05	6.09	5.62	7.76
9D	6.28	5.78	7.96	6.21	5.74	7.61	6.11	5.70	6.67
10	4.35	4.00	7.94	4.21	3.97	5.65	3.72	3.94	−6.09
10A	4.42	4.01	9.23	4.36	3.98	8.54	3.82	3.96	−3.47
10B	4.59	4.25	7.37	4.50	4.22	6.24	4.05	4.19	−3.40
10C	4.64	4.25	8.49	4.55	4.22	7.19	4.47	4.19	6.22
10D	4.69	4.35	7.35	4.62	4.32	6.44	4.57	4.29	6.08
11	14.82	16.09	−8.55	14.53	15.97	−9.93	14.39	15.85	−10.18
11A	15.01	16.09	−7.06	14.72	15.98	−8.50	14.43	15.86	−9.92
11B	15.89	15.28	3.80	15.18	15.27	−0.62	14.44	15.06	−4.32
11C	16.26	15.23	6.34	15.59	15.13	2.99	14.58	15.01	−2.95
11D	16.88	16.44	2.59	16.18	16.33	−0.95	14.82	16.21	−9.34
12	24.65	23.61	4.21	22.58	23.45	−3.86	22.38	23.27	−4.00
12A	25.00	23.99	4.02	22.68	23.83	−5.04	22.55	23.65	−4.89
12B	25.35	24.22	4.45	22.89	24.05	−5.06	22.66	23.87	−5.35
12C	26.22	25.27	3.64	23.47	25.09	−6.91	23.25	24.90	−7.12
12D	26.55	25.98	2.14	23.68	25.80	−8.94	23.55	25.61	−8.71
13	11.44	12.27	−7.30	11.22	12.19	−8.60	10.99	12.10	−10.11
13A	11.61	12.01	−3.38	11.53	11.92	−3.35	11.29	11.83	−4.82
13B	12.17	11.66	4.15	11.95	11.58	3.07	11.33	11.50	−1.48
13C	12.50	12.59	−0.71	12.19	12.50	−2.59	11.94	12.41	−3.91
13D	12.47	11.28	9.52	12.35	11.20	9.27	12.11	11.12	8.20
14	6.64	6.13	7.78	6.55	6.08	7.06	5.98	6.04	−0.94
14A	6.79	6.36	6.93	6.69	6.32	6.24	6.42	6.22	3.11
14B	6.88	6.30	8.51	6.80	6.25	7.98	6.77	6.21	8.30
14C	7.05	6.76	4.08	6.99	6.72	3.84	6.87	6.67	2.93
14D	7.14	6.76	5.22	7.12	6.72	5.62	6.98	6.67	4.52
15	4.79	4.96	−3.66	4.53	4.93	−8.80	4.46	4.89	−9.60
15A	5.09	4.83	5.14	4.89	4.79	1.95	4.53	4.76	−5.06
15B	5.12	4.77	6.81	4.98	4.74	4.82	4.61	4.70	−1.97
15C	5.16	5.37	−4.08	5.13	5.33	−3.85	4.89	5.29	−8.24
15D	5.21	5.36	−2.82	5.20	5.32	−2.28	5.08	5.28	−4.03

The flowability of granular materials is a major consideration in pharmaceutical operations/preparations. High degree of flowability exposes many advantages leading to uniform characterization of the finished products, minimum wear and tear of machine parts and elimination of flows like capping, splitting etc. Therefore this kind of mathematical correlation having small deviation from experimental data within ±10% would be useful in predicting the flow rate of granules. The desired flow rate may be fixed by controlling the variables used in the correlation.
